# How reactions to a brain scan result differ for adults based on self‐identified Black and White race

**DOI:** 10.1002/alz.13558

**Published:** 2023-11-29

**Authors:** Shana D. Stites, Emily A. Largent, Rosalie Schumann, Kristin Harkins, Pamela Sankar, Abba Krieger

**Affiliations:** ^1^ Department of Psychiatry Perelman School of Medicine University of Pennsylvania Philadelphia Pennsylvania USA; ^2^ Department of Medical Ethics and Health Policy Perelman School of Medicine University of Pennsylvania Philadelphia Pennsylvania USA; ^3^ Division of Geriatrics Perelman School of Medicine University of Pennsylvania Philadelphia Pennsylvania USA; ^4^ Department of Statistics Wharton School of Business University of Pennsylvania Philadelphia Pennsylvania USA

**Keywords:** Alzheimer's biomarkers, Alzheimer's stigma, diagnosis confidence, race

## Abstract

**INTRODUCTION:**

How do reactions to a brain scan result differ between Black and White adults? The answer may inform efforts to reduce disparities in Alzheimer's disease (AD) diagnosis and treatment.

**METHODS:**

Self‐identified Black (*n* = 1055) and White (*n* = 1451) adults were randomized to a vignette of a fictional patient at a memory center who was told a brain scan result. Measures of stigma and diagnosis confidence were compared between‐groups.

**RESULTS:**

Black participants reported more stigma than White participants on four of seven domains in reaction to the patient at a memory center visit. Black participants’ confidence in an AD diagnosis informed by a brain scan and other assessments was 72.2 points (95% confidence interval [CI] 70.4 to 73.5), which was lower than the respective rating for White participants [78.1 points (95%CI 77.0 to 79.3)].

**DISCUSSION:**

Equitable access to early AD diagnosis will require public outreach and education that address AD stigma associated with a memory center visit.

## 1 INTRODUCTION

Advances in brain scans and other biomarkers are allowing Alzheimer's disease (AD) diagnosis earlier – even before onset of clinical symptoms. Early diagnosis is essential for increasing benefits from disease‐slowing therapies. Because of known disparities in AD diagnosis rates between Black and White adults,^1^, ^2^ understanding whether Black and White adults react differently to an AD diagnosis informed by a brain scan would be useful to inform efforts aimed at limiting racial disparities in early diagnosis.

Black Americans have long experienced mistreatment and inequity in medicine and research; this includes the inhumane experiments J. Marion Sims conducted on enslaved women in the nineteenth century, intentional withholding of disease‐curing treatment in the twentieth century Tuskegee Study of Untreated Syphilis in the Nego Male, and continues today as biases and barriers^3^ to diagnosis and treatment.^4^, ^5^ The combination of historical and present‐day injustices influence trust in medical care professionals for Black Americans.^6^ Further, they may influence perceptions of medical advances in diagnostic technologies.

A new patient visit at a memory center is a key entry point into the healthcare system for early AD diagnosis. Many factors may contribute to disparities in accessing care at a memory center; one is the public stigma of AD.^7^ Also called AD stigma, it refers to the negative perceptions, attitudes, emotions, and reactions directed at people with AD.^8^, ^9^, ^10^ Black adults may experience greater AD stigma than their White counterparts, given the disproportionately high burden of AD in Black communities.^11^, ^12^, ^13^, ^14^, ^15^, ^16^ That is, a substantial part of AD stigma stems from individuals’ reactions to clinical symptoms,^17^ and Black adults tend to experience a great burden of clinical symptoms.^18^ Understanding how AD stigma differs between Black and White adults in reaction to a new patient visit at a memory center would add to current understanding of AD stigma^17^ and offer novel information about how AD stigma associated with the setting may impact healthcare inequities.

At a memory center, a diagnosis of AD is made by a clinician using data from a clinical history interview, physical exam, and memory tests.^19^ Given that new therapies may be most effective if delivered sooner rather than later,^20^ brain scans, blood tests, and other biomarker testing are likely to be an increasingly important part of diagnosis.^20^, ^21^, ^22^ Brain scans, for example, can measure amyloid and tau burden in the brain, which could, in turn, confirm the presence of targets for emerging therapies. If AD stigma differs between Black and White adults based on a positive versus negative brain scan result, it would offer novel data to understand how expanded use of AD biomarker testing may differentially affect Black and White populations.

The public's confidence in an AD diagnosis may increase when a brain scan or other biomarker test is used in the medical evaluation. Higher confidence in an AD diagnosis would be a benefit of AD biomarkers. With higher confidence in a diagnosis, individuals and their families may focus on addressing care needs and future life planning, rather than seeking out additional clinical evaluations in order to feel confident that they received the correct diagnosis. Given that Black patients are more often misdiagnosed than their White counterparts,^23^, ^24^ they may have lower confidence in an AD diagnosis, particularly when medical tests are used in the evaluation.^25^

The present study compared responses in a sample of 1055 self‐identified Black and 1451 self‐identified White adults. We compare the groups’ AD stigma reactions to a vignette describing a fictional patient at a new patient visit at a memory center. We hypothesize Black adults may have worse AD stigma reactions to a new patient visit at a memory center as compared to White adults based on known healthcare disparities.^23^, ^24^ We also compare the groups’ reactions to the patient being told a positive or negative brain scan result. In addition, we examine how AD diagnosis confidence differed between the groups based on evaluations that varied in number and type of assessment.

**RESEARCH IN CONTEXT**

1. **Systematic review**: Early diagnosis using biomarkers is key for optimizing the benefits of emerging treatments for Alzheimer's disease (AD). Race‐based differences in public reactions to early diagnosis could worsen existing disparities faced by Black Americans. The authors test public reactions between self‐described Black and White adults.
2. **Interpretation**: While a positive AD biomarker test universally causes higher AD stigma, Black adults endorse greater stigma in reaction to a memory center visit and lower confidence in a biomarker‐informed AD diagnosis than their White counterparts.
3. **Future directions**: Education and outreach campaigns that help mitigate stigma associated with specialty AD care may contribute to better equity in access to early diagnosis and treatment. Moreover, scientists also need to understand sociocultural differences in AD diagnosis confidence, its association with diagnosis accuracy and utility.

## 2 METHODS

### 2.1 Study design

This is a vignette‐based experiment. The study flow is shown in Stites et al.^17^ Data collection occurred between June 11 and July 3, 2019.

### 2.2 Setting and participant eligibility

Adults able to read English were invited at random from a large research panel, maintained by Qualtrics. Consenting participants were asked to complete demographic questions. Race was asked using U.S. Census categories. Participants selected all race categories that applied. We classified participants who reported more than one race in a group called “multiple races.” Those who identified as both White and Black alone or in combination with another race were excluded from the study.

The response rate was 53%; the response rate among Black participants was 34% and White participants was 63%. The completion rate was 91.3%, whereby 15.3% of Black participants and 3.8% of White participants discontinued. The sample, which is comprised of 2506 participants, is a combination of individuals who were either in a study sample of the general population^17^ (*n* = 1671) or an oversample of Black or African Americans (*n* = 835).

Participants read a paragraph about AD biomarker testing and then answered a fact‐based comprehension question (Supplemental Material Section A). They were given two opportunities to answer correctly. Participants who failed the second attempt (*n* = 246) were excluded to ensure a minimum level of understanding among participants. About 5.1% of Black participants and 12.6% of White participants failed the[Table alz13558-tbl-0001] screener.

### 2.3 Vignettes

Patient at a memory center visit. All participants read a vignette that described a fictional person who presented for a new patient visit at a memory center with an adult daughter. The vignette stated that the patient answered a “routine set of questions” and underwent “memory testing.” No further information (i.e., interpretation of answers to the questions or results of the memory testing) was provided in the vignette.

The patient in the vignette's race was not specified. We opted a priori to not manipulate the fictional patient's race but rather to use data from this study to inform a future study that will experimentally manipulate multiple signals related to race‐based discrimination. We controlled for patient age at three levels (60, 70, or 80 years old) and gender at two levels (man or woman) to counterbalance effects that could be attributed to these characteristics.

Brain scan test result. The[Table alz13558-tbl-0002] vignette described the patient undergoing a “brain scan test” for an AD biomarker to determine whether the patient's memory problems were caused by AD. Half of participants read a vignette in which the patient learned a positive result and half read about a patient who learned a negative result. The scan result was reported as either “positive” or “negative” for an AD biomarker. This result conforms to U.S. Food and Drug Administration labels for positron emission tomography (PET) biomarker tests that measure brain amyloid. Simple randomization was used to assign participants to a vignette.

The effects of clinical symptom severity of the patient are held constant in our analyses as the number of vignettes were balanced that described symptoms reflecting Clinical Dementia Rating Scale scores of 0 (no symptoms), 1 (mild dementia), or 2 (moderate dementia).^26^ The vignettes were also balanced in terms of whether the doctor in the vignette explained that a treatment was or was not available. Vignette samples are presented in Supplemental Material Section B.

### 2.4 Measures

AD stigma was assessed using a modified Family Stigma in Alzheimer's Disease Scale (FS‐ADS), a validated scale that measures AD stigma across a range of cognitive, emotional, and behavioral attributions.^27^ These attributions align with Link and Phelan's theory of stigma,^28^ the modified labeling theory,^29^ and the social‐cognitive model of stigma.^30^ Items on the original assessment were adapted for relevance to the vignettes.^31^

The modified FS‐ADS is comprised of 41 items that load onto seven empirically‐derived domains. Items asked the extent to which the participant believed that the person described in the vignette: (a) should worry about encountering discrimination by insurance companies or employers and being excluded from voting or medical decision making (Structural Discrimination); (b) would be expected to have certain symptoms like speaking repetitively or not remembering recent events (Negative Severity Attributions); (c) should be expected to have poor hygiene, neglected self‐care, and appear in other ways that provoke negative judgments (Negative Aesthetic Attributions); (d) evoked feelings of disgust or repulsion (Antipathy); (e) would evoke feelings of concern, compassion, or willingness to help from others (Support); (f) would evoke feelings of sympathy, sadness, or pity from others (Pity); and (g) would be ignored or have social relationships limited by others (Social Distance). We framed items on domains that pertained to negative or unpleasant attributes to be about the actions of “others” in order to minimize social desirability bias.^32^, ^33^ Responses were recorded on a 5‐point Likert scale arranged on the screen horizontally from left to right, and analyzed by domain using established methods,^31^ with higher scores indicating stronger endorsement.

AD diagnosis confidence was evaluated using an[Fig alz13558-fig-0001] instrument[Table alz13558-tbl-0003] from Baumann et al..^34^ We modified the diagnostic approaches for relevance in diagnosing AD as follows. Participants rated the level of confidence they would have in an AD diagnosis based on a medical evaluation that included: (a) only a clinical history interview and physical exam; (b) a clinical history interview, physical exam, and memory tests; (c) a clinical history interview, physical exam, memory tests, and blood tests; or (d) a clinical history interview, physical exam, memory tests, blood tests, and a brain scan. Participants rated their confidence for each evaluation on a scale of 0 to 100, with higher scores indicating more confidence.

The Institutional Review Board (IRB) of the {masked for review} reviewed all procedures involving human subjects for the “Health Beliefs Study” (#828348).

### 2.5 Statistical approach

A power calculation using data on the smallest between‐group mean difference on the FS‐ADS and a Type I error rate (alpha) of .05 (two‐sided) showed that a sample of 1200 participants would be sufficient to maintain at least 95% statistical power in estimations of effects.^35^ Means and proportions were used to characterize the sample. Normal 95% confidence intervals (95% CI) and Fisher's exact test of proportions were used to compare the sample to the general population.^36^ ANOVA, Kruskal Wallis, linear regression, and ordered logistic regression (OLR) were used to test for between‐group differences on FS‐ADS domains and Baumann diagnostic confidence items and produced similar results. Common odds ratio (OR) from OLR were used to report association sizes in analysis of the FS‐ADS. Mean differences from linear regression were used to report association sizes in analyses of diagnosis confidence. Bivariate models tested for differences between race groups and between biomarker test results within each race group. Multivariable models statistically control for participant age, gender, Hispanic ethnicity, and educational attainment, which were unbalanced between the race groups. All analyses were balanced for features that varied across vignettes in order to counterbalance effects that could be attributed to them. Statistical tests were two‐sided. *P* values < 0.05 were considered statistically significant. Analyses were performed in Stata 16 (College Station, TX).

## 3 RESULTS

### 3.1 Participant characteristics

The sample of 1055 self‐identified Black and 1451 self‐identified White adults was similar to each other on most assessed characteristics but differed from their respective U.S. general population (Table 1). Black participants were, on average, more likely to be women, Hispanic, and have higher educational attainment than the general Black adult population (all *P* < 0.05). White participants were on average older, less likely to identify with multiple race categories, and more likely to identify as Hispanic than the general White adult population (all *P* < 0.05).

**TABLE 1 alz13558-tbl-0001:** Characteristics of sample and reference populations.

Characteristic	Black participants (*N* = 1055)	U.S. Black adults^b^	White participants (*N* = 1451)	U.S. White adults^b^
Age, mean (95%CI)	38.8 (37.9 to 39.8)	32.0 (31.9 to 32.1)	48.2 (47.3 to 49.1)	41.0 (40.9 to 41.0)
Women, % (95%CI)	53.8 (50.8 to 56.8)	36.3 (36.3 to 36.3)	50.9 (48.3 to 53.4)	50.8 (49.9 to 50.9)
Multiple races,^a^ % (95%CI)	2.2 (1.5 to 3.3)	2.3 (2.3 to 2.3)	1.8 (1.2 to 2.6)	2.8 (2.8 to 2.8)
Hispanic or LatinX	7.3 (5.9 to 9.0)	2.5 (2.5 to 2.5)	15.8 (14.0 to 17.8)	8.7 (8.7 to 8.7)
Education, % (95%CI)				
High school/GED or less	30.3 (27.6 to 33.2)	43. 2 (42.7 to 43.8)	44.0 (41.4 to 46.5)	35.5 (28.2 to 42.7)
Some college or 2‐year degree	40.8 (37.8 to 43.8)	30.3 (29.7 to 30.8)	27.2 (25.0 to 29.6)	28.1 (27.8 to 28.3)
4‐year college degree	19.1 (16.8 to 21.5)	12.0 (11.3 to 12.7)	19.2 (17.2 to 21.3)	18.3 (18.0 to 18.5)
Professional degree	9.9 (8.2 to 11.8)	5.5 (4.9 to 6.3)	9.6 (8.2 to 11.3)	9.4 (9.1 to 9.6)
Known someone with AD,^c^	53.7 (51.1 to 56.3)		48.8 (45.8 to 51.8)	

*Note*: Column percentages may not total 100 due to rounding.

Abbreviations: AD, Alzheimer's disease; CI, confidence interval; GED, general educational development degree; U.S., United States.

^a^ Participants who reported more than one race, excluding those who identified as both White and Black..

^b^ Population data from U.S. Census Bureau.^35^ ^.^

^c^ Percentage responding affirmatively to the question “Do you have, or have you ever had, a person with AD as your relative, friend, or coworker?”

### 3.2 Differences in FS‐ADS scores between Black and White participants toward a patient at a memory center

**FIGURE 1 alz13558-fig-0001:**
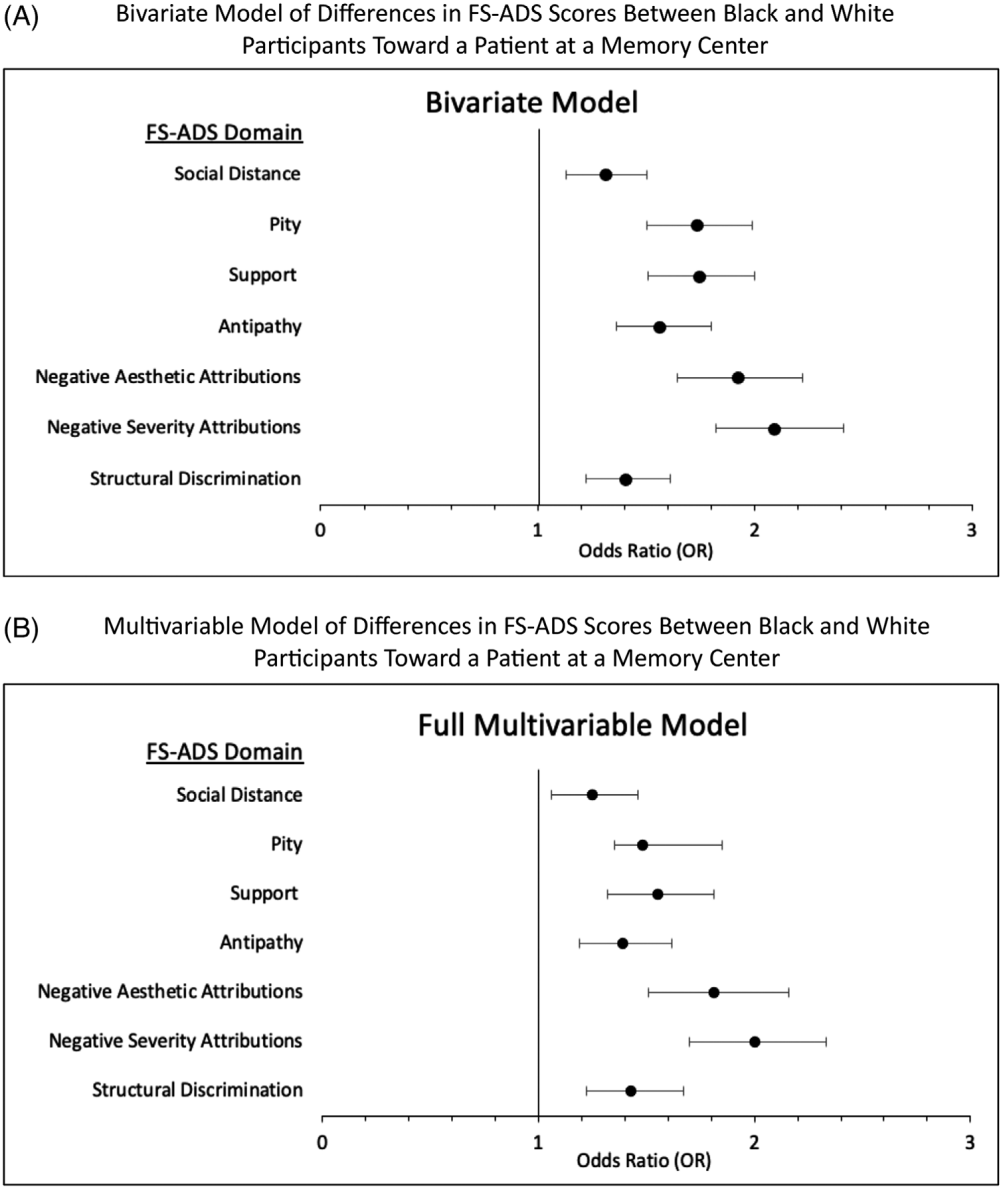
Results of bivariate and multivariable comparisons of Alzheimer's stigma between Black and White participants reacting to a patient at a memory center visit (*N* = 2506). (A) Results of bivariate model of differences in FS‐ADS scores between Black and White participants toward a patient at a memory center. (B) Results of multivariable model of differences in FS‐ADS scores between Black and White participants toward a patient at a memory center. Vertical line marks reference point. 95%CI = normal 95% confidence interval. FS‐ADS = Family Stigma in Alzheimer's Disease Scale. OR = odds ratio from ordered logistic regression. Full model controls for covariates of participant age, gender, Hispanic ethnicity, and educational attainment.

In bivariate comparisons, Black participants endorsed higher FS‐ADS scores than White participants on six of the seven domains: *Structural Discrimination, Negative Severity Attributions, Antipathy, Support, Pity*, and *Social Distance* (Table 2). In multivariable models that statistically adjusted for group differences in age, gender, Hispanic ethnicity, and educational attainment, Black participants endorsed higher scores on *Structural Discrimination* (OR, 1.43, 95%CI 1.22 to 1.67), *Negative Severity Attributions* (OR, 2.00, 95%CI 1.70 to 2.33), *Support* (OR, 1.55, 95%CI 1.32 to 1.81), and *Pity* (OR, 1.48, 95%CI 1.35 to 1.85). Forest plots shown in Figure 1 summarize the results of the bivariate and multivariable comparisons.

**TABLE 2 alz13558-tbl-0002:** Comparisons of Alzheimer's stigma between Black and White participants reacting to a patient at a memory center visit (*N* = 2506).

FS‐ADS domain	Estimate name	Bivariate model	Full model
Structural discrimination	OR (95%CI)	1.40^b^ (1.22 to 1.61)	1.43^b^ (1.22 to 1.67)
	*p*‐value	<0.001	<0.001
Negative severity attributions	OR (95%CI)	2.09^b^ (1.82 to 2.41)	2.00^a^ (1.70 to 2.33)
	*p*‐value	<0.001	01
Negative aesthetic attributions	OR (95%CI)	1.92 (1.64 to 2.22)	1.81 (1.51 to 2.16)
	*p*‐value	88	62
Antipathy	OR (95%CI)	1.56^b^ (1.36 to 1.80)	1.39 (1.19 to 1.62)
	*p*‐value	<0.001	14
Support	OR (95%CI)	1.74^a^ (1.51 to 2.00)	1.55^b^ (1.32 to 1.81)
	*p*‐value	007	001
Pity	OR (95%CI)	1.73^b^ (1.50 to 1.99)	1.48^b^ (1.35 to 1.85)
	*p*‐value	<0.001	<0.001
Social Distance	OR (95%CI)	1.31^b^ (1.13 to 1.50)	1.25 (1.06 to 1.46)
	*p*‐value	<0.001	06

*Note*: Full model controls for covariates of participant age, gender, Hispanic ethnicity, and educational attainment.

Abbreviations: 95%CI, normal 95% confidence interval; FS‐ADS, Family Stigma in Alzheimer's Disease Scale; OR, odds ratio from ordered logistic regression.

^a^ *p* < 0.01.

^b^ *p* < 0.001.

### 3.3 Differences in FS‐ADS scores between Black and White participants in the positive versus negative ad brain scan condition

In bivariate comparisons among Black participants, a positive brain scan result, compared to a negative result, caused higher FS‐ADS scores on six domains: *Structural Discrimination* (OR, 2.62, 95%CI 2.19 to 3.16), *Negative Severity Attributions* (OR, 1.52, 95%CI 1.27 to 1.82), *Antipathy* (OR, 1.55, 95%CI 1.29 to 1.86), *Support* (OR, 1.28, 95%CI 1.07 to 1.54), *Pity* (OR, 2.04, 95%CI 1.70 to 2.44), and *Social Distance* (OR, 1.57, 95%CI 1.30 to 1.89) than a negative test (Table 3). Among White participants, a positive brain scan result, compared to a negative result, caused higher FS‐ADS scores on four domains: *Structural Discrimination* (OR, 2.12, 95%CI 1.72 to 2.64), *Negative Severity Attributions* (OR, 1.28, 95%CI 1.04 to 1.58), *Support* (OR, 1.46, 95%CI 1.18 to 1.80), and *Pity* (OR, 1.78, 95%CI 1.44 to 2.20).

**TABLE 3 alz13558-tbl-0003:** Comparisons of Alzheimer's stigma reactions between Black and White participants in the positive versus negative brain scan test result (*N* = 2506).

		Black	White	Full model
FS‐ADS domain	Estimate name	Positive vs. negative biomarker	Positive vs. negative biomarker	Race group X biomarker result term
Structural discrimination	OR (95% CI)	2.62^b^ (2.19 to 3.16)	2.12^b^ (1.72 to 2.64)	1.17 (.87 to 1.57)
	*p*‐value	<0.001	<0.001	29
Negative severity				
attributions	OR (95% CI)	1.52^b^ (1.27 to 1.82)	1.28^a^ (1.04 to 1.58)	1.06 (.79 to 1.43)
	*p*‐value	<0.001	02	67
Negative aesthetic attributions	OR (95% CI)	1.02 (.83 to 1.26)	1.06 (.84 to 1.33)	93 (.66 to 1.30)
	*p*‐value	84	64	67
Antipathy	OR (95% CI)	1.55^b^ (1.29 to 1.86)	1.15 (.93 to 1.43)	1.28 (.95 to 1.72)
	*p*‐value	<0.001	18	10
Support	OR (95% CI)	1.28^b^ (1.07 to 1.54)	1.46^b^ (1.18 to 1.80)	82 (.61 to 1.10)
	*p*‐value	006	<0.001	18
Pity	OR (95%CI)	2.04^b^ (1.70 to 2.44)	1.78^b^ (1.44 to 2.20)	1.04 (.77 to 1.39)
	*p*‐value	<0.001	<0.001	81
Social distance	OR (95% CI)	1.57^b^ (1.30 to 1.89)	1.21 (.97 to 1.49)	1.28 (.94 to 1.72)
	*p*‐value	<0.001	09	11

*Note*: Full model controls for covariates of participant age, gender, Hispanic ethnicity, and educational attainment.

Abbreviations: 95%CI, normal 95% confidence interval; FS‐ADS, Family Stigma in Alzheimer's Disease Scale; OR, odds ratio from ordered logistic regression.

^a^ *p* < 0.05.

^b^ *p* < 0.001.

In multivariable analyses, no differences were observed in FS‐ADS scores between the positive and negative brain scan test result conditions. These models controlled for group differences in age, gender, Hispanic ethnicity, and educational attainment.

### 3.4 Participant ratings of confidence in an AD diagnosis by evaluation type

Black participants’ confidence in an AD diagnosis that was based on a clinical interview and physical examination was an average rating of 46.8 points (95%CI 45.0 to 48.6), which was higher than the respective rating of White participants [39.2 points (95%CI 37.7 to 40.7), Table 4]. Black participants’ confidence in an AD diagnosis that was based on memory tests in addition to a clinical interview and physical examination was on average rating of 53.6 points (95%CI 51.9 to 55.3), which was also higher than the respective rating for White participants [47.7 points (95%CI 46.3 to 49.1)].

**TABLE 4:** Participant ratings of confidence in an Alzheimer's disease diagnosis by evaluation type.

Evaluation type	Black participants (*n* = 1135) mean (95%CI)	White participants (*n* = 1493) mean (95%CI)	Full model mean difference (95%CI), *p*‐value
Clinical history interview and physical exam only	46.8 (45.0 to 48.6)	39.2 (37.7 to 40.7)	5.6 (3.1 to 8.2), < 0.001
Clinical history interview and physical exam and memory tests	53.6 (51.9 to 55.3)	47.7 (46.3 to 49.1)	4.3 (2.0 to 6.7), < 0.001
Clinical history interview and physical exam, memory tests, and blood tests	60.2 (58.6 to 61.8)	57.2 (55.9 to 58.6)	1.2 (−1.1 to 3.5), .32
Clinical history interview and physical exam, memory tests, and blood tests, and brain scan	72.2 (70.4 to 73.5)	78.1 (77.0 to 79.3)	−6.3 (−8.4 to −4.2), < 0.001

*Note*: Participants were asked to rate their confidence from 0 to 100. Higher values indicate more confidence. Full model controls for covariates of participant age, gender, Hispanic ethnicity, and educational attainment. “How confident would be with your medical evaluation (that is, how the doctor determined what is wrong with you) if the doctor told you that you had a diagnosis of Alzheimer's disease based on a [each ending]?”

Abbreviation: 95%CI, normal 95% confidence interval

**FIGURE 2:**
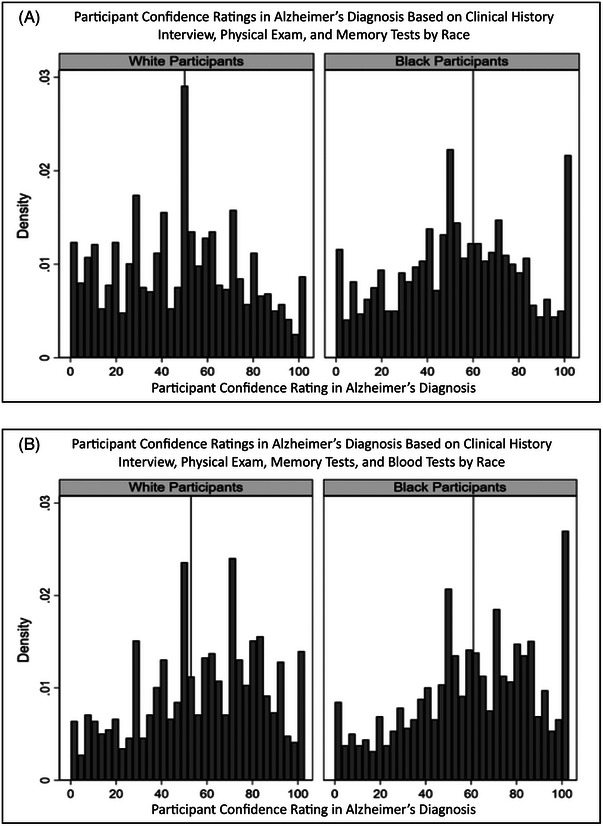
Distribution of confidence ratings in an Alzheimer's diagnosis in White and Black participants (*N* = 2492). Vertical line marks distribution median. (A) Clinical history interview and physical exam and memory tests. (B) Clinical history interview and physical exam, memory tests, and blood tests.

**FIGURE 3:**
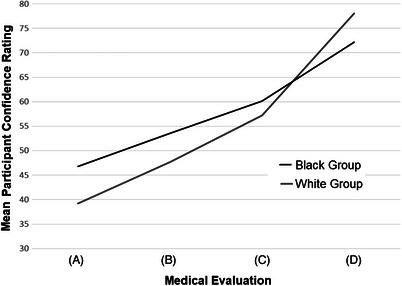
Participant confidence ratings in an Alzheimer's diagnosis by evaluation type. Participants were asked to rate their confidence from 0 to 100. Higher values indicate more confidence. “How confident would be with your medical evaluation (that is, how the doctor determined what is wrong with you) if the doctor told you that you had a diagnosis of Alzheimer's disease based on a [each ending]?”. (A) Clinical history interview and physical exam only. (B) Clinical history interview and physical exam and memory tests. (C) Clinical history interview and physical exam, memory tests, and blood tests. (D) Clinical history interview and physical exam, memory tests, and blood tests, and brain scan.

Black participants’ confidence in an AD diagnosis that was based on blood tests in addition to a clinical interview, physical examination, and memory tests was 60.2 points (95%CI 58.6 to 61.8), which was statistically similar to the respective rating of White participants [57.2 points (95%CI 55.9 to 58.6)]. Black participants’ confidence in an AD diagnosis that was based on a brain scan in addition to the four other assessments was 72.2 points (95%CI 70.4 to 73.5), which was lower than the respective rating for White participants [78.1 points (95%CI 77.0 to 79.3)]. Histograms of participant ratings of AD diagnosis confidence the evaluations that showed the largest and smallest between‐group differences, respectively, are shown in Figure 2. A line graph of mean confidence ratings by group and evaluation type is shown in Figure 3.

## 4 DISCUSSION

Because there are known racial disparities in AD, we conducted a study in a sample of self‐identified Black (*n* = 1055) and White (*n* = 1451) adults. We compared the groups’ AD stigma reactions to a patient at a memory center visit and randomized conditions defined by the patient's brain scan result. We also examined how a participant's confidence in an AD diagnosis was affected by use of a brain scan in an evaluation. We discuss results of each of our three analyses in this section in order to inform efforts to promote racially equitable access to early diagnosis.

First, AD stigma may be a larger barrier for Black adults in accessing memory center care than for White adults. We found support for our hypothesis that Black participants would have worse AD stigma reactions than White participants to a new patient visit at a memory center. In multivariable analyses, Black participants had higher scores on four of seven FS‐ADS domains. These analyses were balanced for biomarker result, severity, and treatment availability and statistically adjusted for group differences in age, gender, Hispanic ethnicity, and educational attainment. The findings suggest that efforts to advance racial equity in AD care may benefit from focusing on destigmatizing memory center care as a key point of access. The findings, consistent with a prior study, also suggest that AD stigma varies in type and intensity across population subgroups.^35^

The largest difference between the race groups was for *Negative Severity Attributions*; Black participants were twice as likely as White participants to attribute greater severity of symptoms to the patient in the vignette (OR, 2.00, 95%CI 1.70 to 2.33). This finding may reflect Black families being more likely to care for family members at home, rather use institutional care.^37^, ^38^ Because of differences in caregiving, they may generally have more experience with individuals who are living with more severe disease compared to their White counterparts. These experiences may lead individuals to associate AD with more severe symptoms. This may contribute to delays in recognizing early symptoms that are less familiar, and, in turn, delay seeking out early diagnosis. In AD, early identification is essential to avoiding some of the most severe consequences of the disease. Population‐level interventions that focus on describing early symptoms and the benefits of early diagnosis may help mitigate negative consequences of the differences in AD stigma between the two race groups.

We also observed a notable difference in structural discrimination: Black participants reported greater worries about structural discrimination than White participants. This finding may be the consequence of historical injustices or contemporary experiences of racism and disparities. Whether injustices occur within or outside memory centers, the artifacts they leave, including worries about recurrent mistreatment, may need to be addressed in memory centers to ensure the pursuit of equity in access and delivery of care.

Second, both race groups reported similar levels of AD stigma due to a positive versus negative brain scan result. We made no formal hypothesis about positive versus negative AD biomarker results. In bivariate comparisons, Black participants showed statistically significant differences in Antipathy (OR, 1.55, 95%CI 1.29 to 1.86) and Social Distance (OR, 1.57, 95%CI 1.30 to 1.89) in response to a positive versus negative biomarker result, whereas White participants did not. These differences observed in the bivariate models were not statistically significant in the multivariable models (discussed in more detail in the next paragraph), which suggests these observed differences may be driven by other factors, such as age and gender, that were correlated with self‐reported race and, potentially, also correlated with disparities in healthcare access.

Patients seeking care at a memory center may fundamentally differ from those within general medical practices or community health clinics. Thus, future studies that investigate how the reactions to a patient seeking AD diagnosis and care vary based on characteristics of the setting may be informative. Such investigations could help identify associations between healthcare disparities and AD stigma, which could in turn aid in guiding efforts aimed at increasing equitable access to diagnosis and care.

In multivariable models that controlled for group differences in age, gender, Hispanic ethnicity, and educational attainment, we examined whether there any aspects of AD stigma – as defined by FS‐ADS domain – that were differentially affected by a positive versus negative AD biomarker result between the two race groups. We found no statistically significant differences. A cautiously optimistic interpretation of this finding is that, while a positive versus negative AD biomarker result may cause more AD stigma, this stigma appears to be similar between the race groups. This should be closely monitored as AD diagnosis is made more accessible to broader ranges of sociodemographic groups. In addition, our finding suggests that prioritizing resources to address aspects of AD stigma that impede access to memory centers, rather than AD biomarkers specifically, may show relatively greater gains in creating racial equity in AD diagnosis and treatment.

Third, differences in confidence in an AD diagnosis varied with the composition of assessments in the medical evaluation. We discovered that Black participants had higher confidence in an AD diagnosis compared to White participants for the first two evaluation types [i.e., (1) clinical history interview and physical exam only and (2) clinical history interview, physical exam and memory tests] but not the second two [i.e., (3) clinical history interview and physical exam, memory tests, and blood tests, and (4) clinical history interview and physical exam, memory tests, and blood tests, and brain scan]. The pattern of findings warrants further study; White participants’ confidence may be more influenced by both the number of tests in an evaluation and the inclusion of biomarker tests (i.e., blood tests and brain scans). Thus, advances in these types of diagnostic methods will not be sufficient to undo racial differences in public skepticism in AD diagnosis, which may be rooted in prior experiences of misdiagnosis.^24^ In fact, an alternative explanation to this pattern of results is that Black participants may have less confidence in examinations that incorporate biomarkers – testing via blood and/or imaging – due to harmful rhetoric surrounding biological differences, as seen, for example, in eugenics.^39^ An emphasis on biological definitions of AD may not be comparably embraced by communities of color as compared to white communities. The reliance on these methods, particularly in the absence of efforts to mitigate racial inequities, may exacerbate healthcare disparities. Studies are needed to understand sociocultural differences in associations among diagnosis confidence and both perceptions of diagnostic accuracy, and usefulness of a diagnosis in planning care and treatment.

While the overall study response rate was 53%, it differed between the two race groups; the response rate among Black participants was about half (34%) that of White participants (63%). Moreover, White participants were more than twice as likely as Black participants to fail the screener (12.6% vs. 5.1%). However, Black participants were four times more likely to discontinue (15.3% vs. 3.8%). The pattern suggests that Black participants were more likely to self‐select out of the study. Those who participated were more educated than the general public (see Table 1). Unfortunately, we do not have data to compare people who did and did not respond to our study. Among those who did respond, language differences may have contributed to some of the difference in why some individuals ended the study early.^17^ Nonetheless, even among this self‐selected, well‐educated group of Black participants, AD stigma – a known barrier to AD care^40^ – was greater than what we observed in the White participant group. This finding underscores the pressing need to actively address barriers and promote equity in access to memory centers and other care settings.

Our study had limitations. Our sample was not representative of the U.S. older adult population. Discrepancies between the participant sample and U.S. population may limit the generalizability of the study results. Future studies with samples representative of more social strata would be useful. In addition, studies that evaluate other aspects of AD diagnosis and care, such as expectations for treatment and qualities of care partners who co‐participate with patients in memory center appointments, would be useful.

Our study used “positive” and “negative” to denote the result of the brain scan. We used this wording as it was consistent with FDA's terms but other descriptions are also used in the field, such as “elevated” and “not elevated”. Future studies that investigate the influence of the choice in wording may be useful. It could be informative to know whether this wording affects individuals’ interpretations or reactions to the result.

## 5 CONCLUSION

A new patient visit at a memory center caused greater stigma for Black participants compared to their White counterparts. This finding was coupled with Black and White participants demonstrating similar reactions to a brain scan result but Black participants expressing lower confidence in an AD diagnosis informed by a brain scan. Our findings suggest equitable access to early AD diagnosis and treatment will require interventions that address AD stigma associated with access to memory center care. Public outreach and education on the use and value of AD biomarkers may be informative for guiding these efforts.

## AUTHOR CONTRIBUTIONS

Shana D. Stites wrote the initial draft of the article.All authors contributed to conceptualizing and writing the article.

## CONFLICT OF INTEREST STATEMENT

The authors have no conflicts to disclose. Author disclosures are available in the supporting information.

## HUMAN PARTICIPANT PROTECTION

The Institutional Review Board of the University of Pennsylvania approved all procedures involving human subjects.

## Supporting information

Supporting Information

Supporting Information
