# Supplementary material for: How reactions to a brain scan result differ for adults based on self‐identified Black and White race
Source: Alzheimers Dement. 2023 Nov 29;20(3):1527–37. doi: 10.1002/alz.13558 (PMC10984417; doi:10.1002/alz.13558)
Supplement: Supplementary file 1 — Supporting Information [file ALZ-20-1527-s002.docx]

Supplemental Materials

Table of Contents

Appendix A – Alzheimer’s Disease Biomarker Comprehension Check

Appendix B – Sample of study vignette

Appendix A

**Alzheimer’s Disease Biomarker Test Comprehension Check**

In this study, you’ll read a story about a patient who is bothered by memory problems, and then you'll answer some questions. The story gives facts about the patient that include the results of their Alzheimer's biomarker tests.

Advances in diagnostic methods can now identify biomarkers of Alzheimer’s disease. Biomarker tests can help detect brain changes and identify people who have biological markers of Alzheimer’s disease. A biomarker measures what is happening inside the living body, shown by the results of laboratory and imaging tests. Biomarkers can help doctors diagnose diseases and health conditions, find health risks in a person, monitor responses to treatment, and see how a person's disease or health condition changes over time. Doctors can measure biomarkers of Alzheimer’s disease before a person has dementia.

[Page Break]

**To confirm that you understood the biomarker information, please select the one statement that is true:**

- Doctors use Alzheimer’s biomarker tests to diagnose the disease.
- A biomarker test is a treatment for Alzheimer’s disease.
- An Alzheimer's biomarker can only be detected in patients who have dementia.

Appendix B

A sample vignette is shown below. More vignettes are available for review in Stites et al.^20^ The underlined text indicates the text that presented variations on the attributes of clinical stage (cognitively normal, mild dementia, *or* moderate dementia), Alzheimer’s biomarker result (memory problems are caused by Alzheimer’s disease, *or* memory problems are not caused by Alzheimer’s disease), and treatment (available *or* not available). The text illustrates how descriptions of treatment and biomarker tests were integrated. In all conditions of either treatment or no treatment, the vignette included text on ways to maintain brain health and follow up (“The doctor advises Mrs. Andrews and her daughter how to promote brain health, including daily physical activity, healthy eating, and the adoption of good sleep habits. The doctor discusses a plan to monitor Mrs. Andrews and schedules her for a follow-up visit.”).

Cognitively Normal, Biomarker Positive, No Treatment

Mr. Andrews is 60 years old. Mr. Andrews comes with his daughter to a memory center for a new patient visit because he is having memory problems and is worried that he might have Alzheimer’s disease. Before beginning the examination, the doctor tells Mr. Andrews that a brain scan test could determine whether his memory problems are being caused by Alzheimer’s disease. But if the problems are caused by Alzheimer’s disease, there is no treatment available that could slow the progression of the disease. Mr. Andrews decides that it would be helpful to have the brain scan.

The doctor begins Mr. Andrews’ examination by asking him a routine set of questions. Mr. Andrews says that he sometimes forgets things, like where he has put his keys. He sometimes struggles to find the right word. It takes him longer now to balance his checkbook. Mr. Andrews says other parts of his life have not changed. He continues to easily follow the evening news. He rarely confuses facts, and it still takes him about the same amount of time to make decisions. He continues to volunteer as a crossing guard for the local elementary school. The doctor also asks Mr. Andrews’ daughter questions. She says that her father usually knows what day it is, never needs to be reminded to take showers, and helps with errands like going to the local post office. To further investigate Mr. Andrews’ cognition, the doctor does a complete examination. This includes a medical history, memory tests, lab tests, and a brain scan.
   Results of Mr. Andrews’ memory testing are normal. His brain scan does show a biomarker for Alzheimer’s disease.
   The doctor explains to Mr. Andrews and his daughter that his memory problems are caused by Alzheimer’s disease and there is no treatment available for Alzheimer’s disease. The doctor advises Mr. Andrews and his daughter how to promote brain health, including daily physical activity, healthy eating, and the adoption of good sleep habits. The doctor discusses a plan to monitor Mr. Andrews and schedules him for a follow-up visit.
